# Left Hemi-Hepatectomy to Resect Metastatic Tumor of Round Ligament of Liver in Patients with Ovarian Cancer

**DOI:** 10.3390/cancers16173036

**Published:** 2024-08-30

**Authors:** Uisuk Kim, Jae Kyung Bae, Junhwan Kim, Ji Hyun Kim, Seong Hoon Kim, Sung-Sik Han, Hyeong Min Park, Sang-Yoon Park, Myong Cheol Lim

**Affiliations:** 1 Center for Gynecologic Cancer, National Cancer Center, Goyang 10408, Republic of Korea; 2 Center for Liver and Pancreatobiliary Cancer, National Cancer Center, Goyang 10408, Republic of Korea; 3 Rare & Paediatric Cancer Branch and Immuno-Oncology Branch, Division of Rare and Refractory Cancer, Research Institute, National Cancer Center, Goyang 10408, Republic of Korea; 4 Department of Cancer Control and Policy, National Cancer Center Graduate School of Cancer Science and Policy, National Cancer Center, Goyang 10408, Republic of Korea

**Keywords:** ovarian cancer, liver metastasis, hemi-hepatectomy, round ligament of liver

## Abstract

**Simple Summary:**

This study suggests that left hemi-hepatectomy should be discussed preoperatively and intraoperatively for the complete resection of a metastatic tumor around the round ligament of the liver. We explain the necessity of left hemi-hepatectomy by the critical vascular anatomy and the pathological report of this study.

**Abstract:**

The objective of this study is to investigate the surgical, clinical and pathological outcomes of left hemi-hepatectomy during cytoreductive surgery (CRS) in patients with primary ovarian cancer. The electronic medical charts of patients with primary ovarian cancer who received CRS including left hemi-hepatectomy from 2000 to 2023 were reviewed and retrospectively analyzed. A total of 17 patients underwent left hemi-hepatectomy for resection of a deep peritoneal implant in the round ligament of the liver during primary CRS. Among these 17 patients, hepatic parenchymal invasion was confirmed in 10 patients (58.8%). Tumor distribution of others is as follows: Glisson’s capsule, hilum, falciform ligament and gall bladder. Fourteen patients (82.4%) achieved CRS; the remaining three patients had residual tumors less than 1 cm. The median period to subsequent chemotherapy was 21 days (range, 12–35 days). No specific complications related to left hepatectomy were identified such as liver failure or bile leakage. Left hemi-hepatectomy for complete surgical resection of a deep peritoneal implant of the round ligament of the liver is surgically feasible and safe.

## 1. Introduction

Peritoneal seeding on the perihepatic area is common in advanced ovarian cancer and it may need extensive upper abdominal surgical procedures including diaphragmatic peritonectomy, cholecystectomy and tumor resection at the porta hepatis or round ligament of the liver [1,2]. Previous studies suggested that these procedures could improve optimal cytoreduction and overall survival in patients with ovarian cancer [3,4,5].

Previous studies have also shown that hepatic parenchymal metastases could originate from two patterns: one is through blood circulation (hematogeneous metastasis) and the other is through peritoneal fluid (transcoleomic seeding) [3,6]. Concealed peritoneal seeding may be found in some locations in the upper abdomen such as the base of the round ligament of the liver, the hepatic fissure, Morison’s pouch and the splenic hilum, as it is a continuation of peritoneal fluid around the liver [7,8,9]. Therefore, hepatic parenchymal invasion through the round ligament of the liver may be deeper than preoperative imaging findings suggest, and left hepatectomy may be required as an intraoperative decision.

Left hemi-hepatectomy refers to the resection of segments 2, 3 and 4 according to the Couinaud classification [10]. Left hemi-hepatectomy is a rare and challenging procedure in ovarian cancer. This is because performing a left hemi-hepatectomy requires understanding the structures around the hepatic fissure, including the left hepatic artery, which are not familiar to gynecologic oncologists [11]. Left hemi-hepatectomy as a part of cytoreductive surgery (CRS) of ovarian cancer has not been investigated specifically. Therefore, in this study, left hemi-hepatectomy as a part of primary CRS is investigated in terms of surgical feasibility, safety and survival outcomes [6,12,13].

## 2. Materials and Methods

### 2.1. Patients and Study Design

From January 2000 to December 2023, 17 patients with primary ovarian cancer received CRS including left hemi-hepatectomy. Data were collected retrospectively from medical records. Surgical and pathological reports were reviewed for all patients.

All patients received a preoperative physical examination and an abdominal and chest CT. Patients underwent left hemi-hepatectomy based on the preoperative CT indicating parenchymal invasion or intraoperative incidental finding of deep invasion of the metastatic tumor. Images and pathological reports were discussed by a tumor board, which is a multidisciplinary team consisting of gynecologic oncologists, radiologists, pathologists, radiation oncologists, medical oncologists, nuclear oncologists and a nursing team. Patients for whom it was not feasible to be optimally debulked according to patients’ general condition or preoperative imaging study results underwent CRS after neoadjuvant chemotherapy.

The extent and resectability of the CRS for the upper abdominal part were determined by the hepatobiliary surgeon by discussing preoperative image or intraoperative findings. All surgical procedures were performed as a team, led by a gynecologic oncologist and in consultation with colorectal, urologic, hepatobiliary and thoracic surgeons. To achieve complete CRS, splenectomy, distal pancreatectomy, cardiophrenic lymphadenectomy, diaphragmatic stripping and/or peritonectomy, urinary tract resection and bowel surgery are included in our center. Hepatic resection was performed following full liver mobilization and diaphragmatic stripping and/or peritonectomy. Complete and optimal CRS was defined as surgery with microscopic residual tumor and less than 1 cm of residual tumor, respectively. All 17 cases including left hemi-hepatectomy were performed by a hepatobiliary surgeon with the assistance of gynecologic oncologists and/or a fellow-in-training.

This retrospective study was approved by the Institutional Review Boards of the institution and conducted in accordance with the principles of the Declaration of Helsinki (National Cancer Center: NCC2022-0075). The requirement for informed consent was waived.

### 2.2. Statistical Analysis

All data were presented as frequencies with percentages for categorical variables and as median and interquartile ranges for continuous variables. Postoperative morbidities were classified according to the Dindo–Clavien classification [14]. Complications were defined as severe that required surgical or radiological intervention (Dindo–Clavien classification ≥ III) and as mild that required only medical treatment (Dindo–Clavien classification I and II).

## 3. Results

### 3.1. Population

During the study period, 17 patients with advanced ovarian cancer who underwent primary CRS including left hemi-hepatectomy were identified. The median patient age was 58 years (range, 44–69 years). Serous adenocarcinoma was the most common histologic type (14 patients, 82.4%). Endometrioid, clear cell, and carcinosarcoma were each identified once (5.9%). Of the 17 patients, 15 (88.2%) had FIGO stage IVB, 7 patients (41.2%) had grade 2 tumors and 10 patients (58.8%) had grade 3 tumors. The median preoperative serum CA-125 level was 1234 (range, 38–20,351). Of the 17 patients, 12 (70.6%) patients underwent upfront primary CRS and others underwent interval CRS, which means CRS after neoadjuvant chemotherapy. Underlying comorbidities of patients are as follows: Four patients (23.5%) had hypertension, three patients (17.6%) had diabetes mellitus and two patients (11.8%) had tuberculosis before the surgery. One patient had cardiovascular disease and two patients had dyslipidemia. Because the number of patients with underlying diseases was small, there were no differences in underlying conditions based on the timing of surgery. For three patients (17.6%), direct hepatic parenchymal invasion through the round ligament of the liver was observed in preoperative imaging (CT or PET-CT), while the remaining fourteen patients (82.4%) showed no definite hepatic parenchymal invasion through the round ligament (Table 1).

### 3.2. Surgical Procedures

Table 2 details additional surgical procedures combined with left hemi-hepatectomy during CRS. Fifteen patients (88%) underwent hysterectomy whereas the other two patients had prior hysterectomy due to myoma uteri. All patients underwent salpingo-oophorectomy and para-aortic lymphadenectomy. Pelvic lymphadenectomy and supracolic omentectomy were each performed in 13 patients (76%). The majority of patients received other major upper abdominal procedures combined with left hemi-hepatectomy, including splenectomy (12 patients, 71%), distal pancreatectomy (4 patients, 24%), cholecystectomy (12 patients, 71%) and diaphragmatic peritonectomy (10 patients, 59%). Also, colorectal surgical procedures were performed in some patients. Low anterior resection and appendectomy were performed in seven (41%) and eight (47%) patients, respectively.

### 3.3. Surgical and Pathological Outcomes

In Table 3, surgical outcomes are presented. The median operative time was 507 minutes (range, 311–760 min) and the median estimated blood loss was 900 mL (interquartile range, 700–1375 mL). The median duration of postoperative intensive care unit stay was 1 day (range, 0–2 days) and the median hospitalization after operation was 14 days (range 8–27 days). The median time to subsequent chemotherapy was 21 days (range 12–35 days). Chemotherapy regimens were as follows: twelve patients received paclitaxel-carboplatin chemotherapy, one patient received paclitaxel-carboplatin-bevacizumab, and the remaining four patients received paclitaxel-carboplatin-oregovomab. Fourteen patients (82.4%) resulted in surgery with a microscopic residual tumor. The other three patients had a residual mass less than 1 cm (17.6%) in the small bowel mesentery. Tumor invasion was confirmed in 16 patients (94.1%) and their distributions are as follows: liver parenchyma (10, 58.8%), Glisson’s capsule or hilum (14, 82.4%), falciform ligament (11, 64.7%) and gall bladder (5, 29.4%). Among them, hepatic parenchymal invasion was pathologically confirmed in 10 patients (58.8%). The tumor size in the left hemi-hepatectomy specimen varied among 10 patients (Supplementary Table S1). No tumor remained in one patient who received three cycles of neoadjuvant chemotherapy before surgery.

### 3.4. Morbidities

Postoperative morbidities are presented in Table 4. A severe complication is defined as Dindo classification grade 3 or higher, requiring surgical or radiological intervention, while a mild complication corresponds to Dindo classification grades 1 and 2, requiring only medical treatment [15]. One patient had gastrointestinal bleeding as hematemesis due to a postoperative stress ulcer and was treated by embolization of the left gastric artery and left inferior phrenic artery. One patient with significant intraperitoneal subhepatic bleeding was managed by blood transfusion. Twelve patients (71%) were successfully treated medically as follows: Two patients with infection were treated with antibiotics. One patient had a wound infection with methicillin-resistant Staphylococcus aureus (MRSA) and was treated with antibiotics, while another patient had a Jackson–Pratt drain site infection with MRSA and also received antibiotic treatment. Five patients had ileus and were managed by nasogastric tube insertion. Four patients developed postoperative thromboembolism; they were stable in their vital signs and managed with anticoagulants. No complications related to major hepatic surgery were identified such as liver failure or bile leakage [16,17]. Of the 17 surgeries, the 12-month survival rate is 93.7% (15/16). One patient died due to neutropenic shock complicated by urosepsis.

## 4. Discussion

In this study, we analyzed patients with primary ovarian cancer receiving CRS including left hemi-hepatectomy. Among 17 patients, hepatic parenchymal invasion was confirmed in 10 patients (58.8%) by pathological report after left hemi-hepatectomy. All patients achieved optimal cytoreduction, with complete cytoreduction in 14 patients (82.4%). Complication after surgery was medically managed except in one case that required radiological embolization of arteries, due to gastrointestinal tract bleeding.

**Figure 1:**
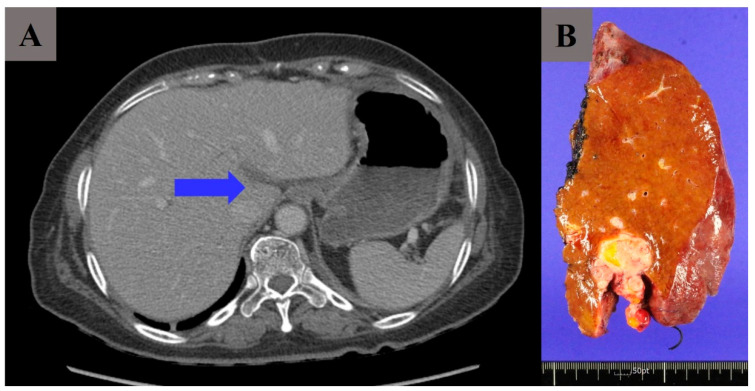
(**A**) A tumor at the hepatic fissure (arrow) (round ligament of liver). (**B**) Surgical specimen of left hemi-hepatectomy. (**A**,**B**) are of the same patient.

Excluding three patients (17.6%), hepatic involvement was not observed in the preoperative imaging of the remaining fourteen patients. However, tumor invasion through the round ligament of the liver was suspected intraoperatively in those patients, and left hemi-hepatectomy was performed by a hepatobiliary surgeon. It could be found in one of our cases (Figure 1). In preoperative imaging, the tumor was identified as being limited to the round ligament, not invading the hepatic parenchyma (Figure 1A). However, intraoperatively, it was identified that the tumor invasion was deeper than the preoperative image findings showed along the falciform ligament, which made left hemi-hepatectomy the inevitable choice (Figure 1B). The invasion of liver segment 4 was pathologically confirmed. Importantly, left hemi-hepatectomy is more often decided intraoperatively because deep sheet-like invasion of the tumor is found during surgery, rather than predicted preoperatively.

In the aspect of peritoneal fluid circulation, peritoneal fluid flows to the right upper quadrant from the right paracolic gutter due to pressure differences. This leads to the accumulation of peritoneal fluid in the right upper abdomen; therefore, peritoneal seeding of the subphrenic area is common along its convex surface [18]. The perihepatic space includes the falciform ligament, gall bladder fossa and Morison’s pouch, which all communicate with each other through peritoneal fluid; therefore, gynecologic oncologists should be aware of the wide and deep invasion of peritoneal seeding [19,20].

Ovarian cancer metastasizes along the peritoneum, and intrahepatic peritoneal seeding along the round ligament of the liver could only be identified intraoperatively after meticulous dissection by hepatobiliary surgeons. However, it is not easy to assess the exact extent of intrahepatic invasion through the round ligament based on preoperative imaging. In fact, in ovarian cancer, small volumes of peritoneal carcinomatosis in the abdomen are not all detectable by CT [21]. Especially for the right upper quadrant, it is well known by radiologists that falciform ligament invasion and the subphrenic area should be reviewed in multiple planes to differentiate from liver parenchymal deposit [22,23,24].

**Figure 2:**
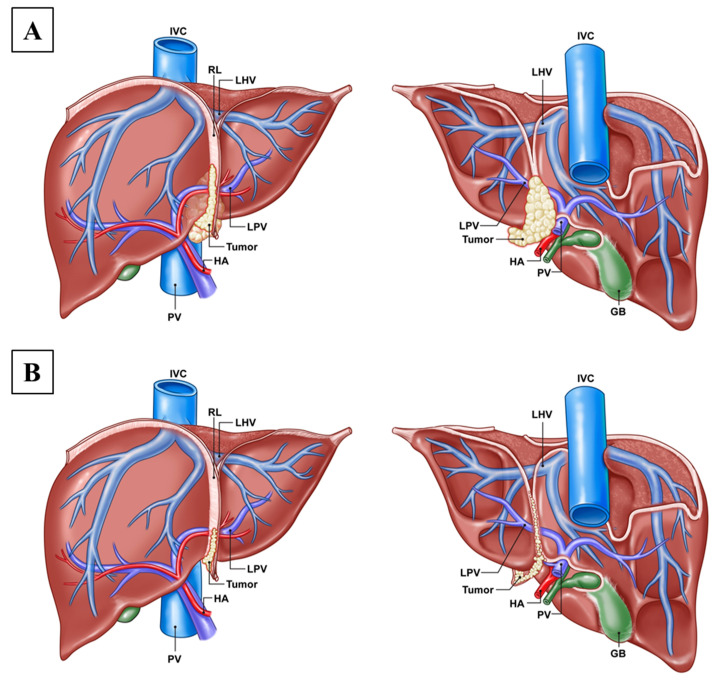
(**A**) Anterior/posterior view of the liver: a bulky tumor (yellow) presented around the round ligament of the liver. It can be easily detected by imaging and hepatic invasion is often suspected. We could decide on performing left hemi-hepatectomy before surgery. (**B**) Anterior/posterior view of the liver: tumor (yellow) spread thinly along the course of the round ligament, downward and backward of the liver. It might appear like a sheath or sand on a beach, making it difficult to determine the exact extent of the tumor by preoperative imaging. We should discuss whether to perform a left hemi-hepatectomy for complete tumor resection intraoperatively. IVC, inferior vena cava; RL, round ligament of liver; LHV, left hepatic vein; PV, portal vein; LPV, left portal vein; HA, hepatic artery; GB, gall bladder.

Tumor metastasis in this area increases the complexity of surgery because the hepatic vessels to the left and right hepatic lobes branch off near this area. The round ligament of the liver is an anatomical landmark in hepatic surgery. It is a thin fold of peritoneum that attaches to the subdiaphragmatic surface and runs downward anteriorly between the right and left lobes of the liver [25,26]. It is continuous with the ligament on both sides and backward to the gall bladder and portal triad [14]. A previous study also demonstrated the peritoneal metastases patterns of the liver area in ovarian cancer. One is superficial metastasis involving Glisson’s sheet with no parenchymal infiltration and the other is carcinomatosis along the lines between the liver and surrounding ligaments [27]. A bulky tumor around the round ligament can be easily detected by imaging, and in such cases, hepatic invasion is often suspected. We could decide on performing left hemi-hepatectomy before surgery (Figure 2A). Peritoneal seeding may spread thinly along the course of the round ligament, downward and backward of the liver. It might appear like a sheath or sand on a beach, making it difficult to determine the exact extent of the tumor through imaging. We should discuss whether to perform a left hemi-hepatectomy for complete tumor resection intraoperatively (Figure 2B).

One of the surgical considerations in this area is that with tumor invasion along the round ligament, especially just behind the upper base of the round ligament of the liver, the middle and left hepatic veins both drain into the inferior vena cava [28]. As seen from the inferior view of the liver, the ligamentum teres hepatis, which is the lower edge of the round ligament and the remnant of the umbilical vein, joins the left branch of the portal vein. Therefore, if a deep peritoneal implant of the round ligament of the liver is suspected, the ligation of the hepatic vein or portal vein and performing left hemi-hepatectomy are inevitable for complete tumor resection in this area and to achieve margin-free resection (Figure 2). In this study, hepatic parenchymal invasion was pathologically confirmed in more than half of patients requiring left hemi-hepatectomy (10 patients, 58.8%). All 17 patients were suspected of tumor infiltration around the round ligament of the liver. To explore the extent of cancer involvement at the base of the round ligament, left hemi-hepatectomy was deemed necessary and was performed intraoperatively. The cancer around the round ligament could infiltrate into the liver parenchyma or spread via the peritoneum, leading to capsule invasion.

No specific complications related to major hepatic surgery were identified such as liver failure or bile leakage [16,17]. In this study, most of the patients were successfully managed with medical treatment and only one patient required radiologic embolization due to gastrointestinal tract bleeding followed by a stress ulcer. The median period to subsequent chemotherapy was 21 days (range, 12–35 days) in this study, which is comparable to that of postoperative chemotherapy for ovarian cancer patients reported in previous studies (interquartile range, 23–43 days) [29,30]. This suggests that CRS including left hemi-hepatectomy is feasible and safe from a surgical perspective, with acceptable morbidity when performed on suitable patients.

This study was retrospective and there are some missing data due to the use of medical records. The number of patients is small because left hemi-hepatectomy as part of primary CRS for ovarian cancer is rare. The surgical decision for left hemi-hepatectomy had been determined intraoperatively in most cases. However, the surgical procedures in the operating room cannot be precisely predicted and a retrospective approach was justified. Further studies are required to confirm the findings of this study and to analyze the survival benefits of this surgical procedure. A prospective study can be attempted by comparing preoperative imaging that suggests hepatic parenchymal metastasis or seeding nodules in the perihepatic area with the actual findings observed during surgery. Furthermore, to enhance the accuracy of assessing disease extent, which in turn improves the prediction of complete cytoreduction, the addition of 18F-FDG-PET to contrast-enhanced CT plays an expanding role in improving precision. In recurrent ovarian cancer, a previous study demonstrated that PET/CT (sensitivity, 91%; specificity, 88%) is more accurate than CT (sensitivity, 79%; specificity, 84%) or MRI (sensitivity, 75%; specificity, 78%). Even in cases of primary ovarian cancer like in this study, FDG-PET could be effectively further utilized to accurately evaluate the extent of the cancer [31,32,33,34,35,36,37].

This is the first study focusing on left hemi-hepatectomy as part of primary CRS for ovarian cancer with the review of anatomical consideration of adjacent critical vessels along with the round ligament of the liver and it may further advance optimal cytoreductive surgery in patients with ovarian cancer.

## 5. Conclusions

In conclusion, we suggest that left hemi-hepatectomy should be discussed preoperatively and intraoperatively for complete tumor resection around the round ligament of the liver in selected cases. This study highlights that peritoneal seeding may spread along the round ligament, leading to hepatic infiltration in nearly half of cases. Given the critical vascular anatomy and pathological findings, left hemi-hepatectomy is feasible with acceptable morbidity and may enhance optimal cytoreduction in ovarian cancer patients.

## Figures and Tables

**Table 1:** Patient characteristics.

Variables	No. [Range] (%)
Age at diagnosis (year)	58 [44–69]
Histologic type	
Serous adenocarcinoma	14 (82.4%)
Endometrioid	1 (5.9%)
Clear cell	1 (5.9%)
Carcinosarcoma	1 (5.9%)
Tumor grade	
2	7 (41.2%)
3	10 (58.8%)
FIGO stage	
IIIB	1 (5.9%)
IIIC	1 (5.9%)
IVB	15 (88.2%)
Preoperative serum CA-125 (IU/ml)	1234 [38–20,351]
The timing of CRS ^1^	
Upfront CRS	12 (70.6%)
Interval CRS	5 (29.4%)
Comorbidities	
Hypertension	4 (23.5%)
Diabetes Mellitus	3 (17.6%)
Cardiovascular disease	1 (5.9%)
Tuberculosis	2 (11.8%)
Liver parenchymal invasion by preoperative imaging	
Yes	3 (17.6%)
No	14 (82.4%)

Values are presented as number (%) or median [range]. ^1^ CRS, cytoreductive surgery.

**Table 2:** Additional surgical procedures combined with left hemi-hepatectomy during cytoreductive surgery.

Type of Procedures	No. (%)
Hysterectomy	15 (88%)
Salpingo-oophorectomy	17 (100%)
Pelvic lymphadenectomy	13 (76%)
Para-aortic lymphadenectomy	17 (100%)
Supracolic omentectomy	13 (76%)
Splenectomy	12 (71%)
Distal pancreatectomy	4 (24%)
Cholecystectomy	12 (71%)
Diaphragmatic peritonectomy	10 (59%)
Low anterior resection	7 (41%)
Appendectomy	8 (47%)

Values are presented as numbers (%).

**Table 3:** Surgical outcomes and pathologically proven tumor involvement.

Surgical Outcome	No. [Range] (%)
Operative time (min)	507 [311–760]
Estimated blood loss (mL)	900 [500–3600]
Postoperative ICU ^1^ stay (day)	1 [0–2]
Hospitalization after operation (day)	14 [8–27]
Time to subsequent chemotherapy (day)	21 [12–35]
Residual disease	
Microscopic	14 (82.4%)
Macroscopic < 1 cm	3 (17.6%)
Pathologically Proven Tumor Involvement	No. (%)
No tumor remained	1 (5.9%)
Liver parenchyma	10 (58.8%)
Glisson’s Capsule or hilum	14 (82.4%)
Falciform ligament	11 (64.7%)
Gall bladder	5 (29.4%)

Values are presented as numbers (%) or medians [range]. ^1^ ICU, intensive care unit.

**Table 4:** Morbidities after cytoreductive surgery including left hemi-hepatectomy.

Variables	No. (%)	Management
Severe complication ^1^		
Gastrointestinal tract bleeding (stress ulcer)	1 (6%)	Embolization
Mild complication ^2^		
Infection	2 (12%)	Antibiotics
Ileus	5 (29%)	Nasogastric tube insertion
Thromboembolism	4 (24%)	Anticoagulants
Subhepatic hemorrhage/hematoma	1 (6%)	Transfusion and observation

Values are presented as numbers (%). ^1^ Severe complication: required surgical/radiological intervention. ^2^ Mild complication: required medical treatment.

## Data Availability

The data from are available on reasonable request to the corresponding author.

## Supplementary Materials

The following supporting information can be downloaded at: https://www.mdpi.com/article/10.3390/cancers16173036/s1, Table S1: Tumor size in the left hemi-hepatectomy specimen.

## References

[1] Tozzi R., Traill Z., Campanile R.G., Ferrari F., Majd H.S., Nieuwstad J., Hardern K., Gubbala K. (2016). Porta hepatis peritonectomy and hepato–celiac lymphadenectomy in patients with stage IIIC–IV ovarian cancer: Diagnostic pathway, surgical technique and outcomes. Gynecol. Oncol..

[2] Eisenhauer E.L., Abu-Rustum N.R., Sonoda Y., Levine D.A., Poynor E.A., Aghajanian C., Jarnagin W.R., DeMatteo R.P., D’Angelica M.I., Barakat R.R. (2006). The addition of extensive upper abdominal surgery to achieve optimal cytoreduction improves survival in patients with stages IIIC–IV epithelial ovarian cancer. Gynecol. Oncol..

[3] Lim M.C., Kang S., Lee K.S., Han S.S., Park S.J., Seo S.S., Park S.Y. (2009). The clinical significance of hepatic parenchymal metastasis in patients with primary epithelial ovarian cancer. Gynecol. Oncol..

[4] Chi D.S., Franklin C.C., Levine D.A., Akselrod F., Sabbatini P., Jarnagin W.R., DeMatteo R., Poynor E.A., Abu-Rustum N.R., Barakat R.R. (2004). Improved optimal cytoreduction rates for stages IIIC and IV epithelial ovarian, fallopian tube, and primary peritoneal cancer: A change in surgical approach. Gynecol. Oncol..

[5] Song Y.J., Lim M.C., Kang S., Seo S.-S., Park J.W., Choi H.S., Park S.-Y. (2009). Total colectomy as part of primary cytoreductive surgery in advanced Müllerian cancer. Gynecol. Oncol..

[6] Bacalbasa N., Dima S., Brasoveanu V., David L., Balescu I., Purnichescu-Purtan R., Popescu I. (2015). Liver resection for ovarian cancer liver metastases as part of cytoreductive surgery is safe and may bring survival benefit. World J. Surg. Oncol..

[7] Kostov S., Selçuk I., Watrowski R., Dineva S., Kornovski Y., Slavchev S., Ivanova Y., Yordanov A. (2024). Neglected Anatomical Areas in Ovarian Cancer: Significance for Optimal Debulking Surgery. Cancers.

[8] O’Neill A.C., Somarouthu B., Tirumani S.H., Braschi-Amirfarzan M., Van den Abbeele A.D., Ramaiya N.H., Shinagare A.B. (2017). Patterns and prognostic importance of hepatic involvement in patients with serous ovarian cancer: A single-institution experience with 244 patients. Radiology.

[9] Eisenkop S.M., Spirtos N.M., Lin W.-C.M. (2006). Splenectomy in the context of primary cytoreductive operations for advanced epithelial ovarian cancer. Gynecol. Oncol..

[10] Couinaud C. (1999). Liver anatomy: Portal (and suprahepatic) or biliary segmentation. Dig. Surg..

[11] Gasparri M.L., Grandi G., Bolla D., Gloor B., Imboden S., Panici P.B., Mueller M.D., Papadia A. (2016). Hepatic resection during cytoreductive surgery for primary or recurrent epithelial ovarian cancer. J. Cancer Res. Clin. Oncol..

[12] Luna-Abanto J., García Ruiz L., Laura Martinez J., Álvarez Larraondo M., Villoslada Terrones V. (2020). Liver resection as part of cytoreductive surgery for ovarian cancer. J. Gynecol. Surg..

[13] Merideth M.A., Cliby W.A., Keeney G.L., Lesnick T.G., Nagorney D.M., Podratz K.C. (2003). Hepatic resection for metachronous metastases from ovarian carcinoma. Gynecol. Oncol..

[14] Garbar V., Newton B.W. (2019). Anatomy, Abdomen and Pelvis, Falciform Ligament.

[15] Dindo D. (2014). The Clavien–Dindo classification of surgical complications. Treatment of Postoperative Complications after Digestive Surgery.

[16] Li B., Qin Y., Qiu Z., Ji J., Jiang X. (2021). A cohort study of hepatectomy-related complications and prediction model for postoperative liver failure after major liver resection in 1,441 patients without obstructive jaundice. Ann. Transl. Med..

[17] Chi D.S., Zivanovic O., Levinson K.L., Kolev V., Huh J., Dottino J., Gardner G.J., Leitao M.M., Levine D.A., Sonoda Y. (2010). The incidence of major complications after the performance of extensive upper abdominal surgical procedures during primary cytoreduction of advanced ovarian, tubal, and peritoneal carcinomas. Gynecol. Oncol..

[18] Suh D.H., Kim N.K., Kim K.D., No J.H., Kim Y.B. (2022). EP243/# 1167 Relative extensiveness of peritoneal seeding versus lymph node metastasis as a prognostic factor in advanced-stage ovarian cancer. Int. J. Gynecol. Cancer.

[19] Turco G., Chiesa G., de Manzoni G. (1988). Echographic anatomy of the greater peritoneal cavity and its recesses. La Radiol. Med..

[20] Chandrashekhara S.H., Triveni G.S., Kumar R. (2016). Imaging of peritoneal deposits in ovarian cancer: A pictorial review. World J. Radiol..

[21] Salani R., Axtell A., Gerardi M., Holschneider C., Bristow R.E. (2008). Limited utility of conventional criteria for predicting unresectable disease in patients with advanced stage epithelial ovarian cancer. Gynecol. Oncol..

[22] Zorzetto G., Coppola A., Molinelli V., Angeretti M.G., Casarin J., Fontana F., Piacentino F., Carcano G., Ghezzi F., Venturini M. (2022). Spectral CT in peritoneal carcinomatosis from ovarian cancer: A tool for differential diagnosis of small nodules?. Eur. Radiol. Exp..

[23] Avesani G., Arshad M., Lu H., Fotopoulou C., Cannone F., Melotti R., Aboagye E., Rockall A. (2020). Radiological assessment of Peritoneal Cancer Index on preoperative CT in ovarian cancer is related to surgical outcome and survival. La Radiol. Med..

[24] Iyer V.R., Lee S.I. (2010). MRI, CT, and PET/CT for ovarian cancer detection and adnexal lesion characterization. Am. J. Roentgenol..

[25] Selçuk İ., Başarır Z.Ö., Ohri N., Akar B., Çalışkan E., Güngör T. (2019). Comparative surgical resection of the ligamentum teres hepatis in a cadaveric model and a patient with ovarian cancer. J. Turk. Ger. Gynecol. Assoc..

[26] Cavaness K.M., Chapman W.C. (2019). Management of Metastatic Colorectal Cancer to the Liver. Shackelford’s Surgery of the Alimentary Tract, 2 Volume Set.

[27] Rosati A., De Rose A.M., Gallotta V., Giannarelli D., Ghirardi V., Pavone M., De Palma A., Conte C., Marchetti C., Gallucci V. (2024). Feasibility and operative outcomes of surgery in the liver area in advanced ovarian cancer. Gynecol. Oncol..

[28] Ibukuro K., Fukuda H., Tobe K., Akita K., Takeguchi T. (2016). The vascular anatomy of the ligaments of the liver: Gross anatomy, imaging and clinical applications. Br. J. Radiol..

[29] Lin H., Chen W.-H., Wu C.-H., Ou Y.-C., Chen Y.-J., Chen Y.-Y., Lin Y.-H., Fu H.-C. (2021). Impact of the time interval between primary debulking surgery and start of adjuvant chemotherapy in advanced epithelial ovarian cancer. Cancer Manag. Res..

[30] Liu X., Zhao Y., Jiao X., Yu Y., Li R., Zeng S., Chi J., Ma G., Huo Y., Li M. (2023). Timing of interval debulking surgery and postoperative chemotherapy after neoadjuvant chemotherapy in advanced epithelial ovarian cancer: A multicenter real-world study. J. Ovar. Res..

[31] Schwarz J.K., Grigsby P.W., Dehdashti F., Delbeke D. (2009). The role of 18F-FDG PET in assessing therapy response in cancer of the cervix and ovaries. J. Nucl. Med..

[32] Kitajima K., Murakami K., Yamasaki E., Kaji Y., Fukasawa I., Inaba N., Sugimura K. (2008). Diagnostic accuracy of integrated FDG-PET/contrast-enhanced CT in staging ovarian cancer: Comparison with enhanced CT. Eur. J. Nucl. Med. Mol. Imaging.

[33] Javitt M.C. (2007). ACR Appropriateness Criteria® on Staging and Follow-Up of Ovarian Cancer. J. Am. Coll. Radiol..

[34] Yoshida Y., Kurokawa T., Kawahara K., Tsuchida T., Okazawa H., Fujibayashi Y., Yonekura Y., Kotsuji F. (2004). Incremental benefits of FDG positron emission tomography over CT alone for the preoperative staging of ovarian cancer. Am. J. Roentgenol..

[35] Sebastian S., Lee S.I., Horowitz N.S., Scott J.A., Fischman A.J., Simeone J.F., Fuller A.F., Hahn P.F. (2008). PET–CT vs. CT alone in ovarian cancer recurrence. Abdom. Imaging.

[36] Ea H. (2005). Evaluation of integrated whole-body PET/CT in the detection of recurrent ovarian cancer. Eur. J. Radiol..

[37] Gu P., Pan L.-L., Wu S.-Q., Sun L., Huang G. (2009). CA 125, PET alone, PET–CT, CT and MRI in diagnosing recurrent ovarian carcinoma: A systematic review and meta-analysis. Eur. J. Radiol..

