# Supplementary material for: Left Hemi-Hepatectomy to Resect Metastatic Tumor of Round Ligament of Liver in Patients with Ovarian Cancer
Source: Cancers (Basel). 2024 Aug 30;16(17):3036. doi: 10.3390/cancers16173036 (PMC11394477; doi:10.3390/cancers16173036)
Supplement: Supplementary file 1 [file cancers-16-03036-s001.zip › cancers-3172304-supplementary.pdf]

Supplementary Table S1. Tumor size in the left hemi-hepatectomy specimen

**Case**	**dimension (cm)**
1	1.7 x 1.0
2	1.0 x 0.8
3	2.0 x 1.6
4	8.0 x 5.5
5	2.0 x 1.5
6	8.0 x 7.7
7	3.5 x 1.0
8	2.0 x 1.8
9	4.8 x 3.2
10	1.1 x 0.7
